# Electrochemical Detection of Neuronal Injury in Cell Culture Samples: A Cost-Effective Biosensor for Neurofilament Light Sensing

**DOI:** 10.3390/bios16040212

**Published:** 2026-04-09

**Authors:** Anna Panteleeva, Sujey Palma-Florez, Ashlyne M. Smith, Sara Palma-Tortosa, Zaal Kokaia, Josep Samitier, Mònica Mir

**Affiliations:** 1 Nanobioengineering Group, Institute for Bioengineering of Catalonia (IBEC), Barcelona Institute of Science and Technology (BIST), 08028 Barcelona, Spain; palmaflorezsujey@gmail.com (S.P.-F.); ashlynesmith@utexas.edu (A.M.S.); jsamitier@ibecbarcelona.eu (J.S.); 2 Department of Electronics and Biomedical Engineering, University of Barcelona, 08007 Barcelona, Spain; 3 Laboratory of Stem Cells and Restorative Neurology, Lund Stem Cell Center, Lund University, 221 84 Lund, Sweden; sara.palma_tortosa@med.lu.se (S.P.-T.); zaal.kokaia@med.lu.se (Z.K.); 4 Biomedical Research Networking Center in Bioengineering, Biomaterials and Nanomedicine (CIBER-BBN), 28029 Madrid, Spain

**Keywords:** neurofilament light chain (NfL), electrochemical immunosensor, label-free detection, cell culture matrix, human-derived neurons, biosensor, in vitro neuronal injury model

## Abstract

Neurofilament light chain (NfL) is a promising biomarker of axonal injury across acute and chronic neurodegeneration, which can improve drug discovery and disease monitoring models. Traditional in vivo animal models cannot fully mimic human pathophysiology of neurodegenerative diseases (NDDs), but in vitro models based on human cells solve this problem, reducing the time and cost of drug testing. We developed an electrochemical immunosensor for NfL detection in cell culture media to monitor acute neuronal injury in in vitro models. The biosensor was designed in two configurations: the label-free system, which directly detects NfL in the sample via the antibody–antigen interaction, and the sandwich configuration, which incorporates two additional antibodies. Detection was examined using electrochemical techniques, including cyclic voltammetry (CV), electrochemical impedance spectroscopy (EIS), and chronoamperometry (CA). The sensor demonstrated a detection limit of 3–9 pg mL^−1^, and a dynamic working range spanning from 10 up to 10^7^ pg mL^−1^. Importantly, NfL was successfully detected in physiological media collected from cultured neurons that were differentiated from the long-term human neuroepithelial-like stem cells. This discovery highlights the platform’s applicability for in vitro neurodegenerative models. The immunosensor offers a sensitive, scalable, and cost-effective alternative for neurodegeneration detection in drug testing applications.

## 1. Introduction

Neurodegenerative diseases (NDDs) affect over 50 million individuals worldwide, impairing their cognitive and motor abilities and leading to significant declines in quality of life. These diseases, namely Alzheimer’s disease (AD), are characterised by the progressive degeneration and death of neurons, which are the fundamental building blocks of the brain and spinal cord [1]. As a result, affected individuals often experience a range of symptoms, including memory loss, reduced movement, altered behaviour, and a decline in their ability to carry out everyday tasks. The prevalence of NDDs has been steadily rising in recent years, posing a substantial public health challenge globally [2].

While our understanding of NDDs has advanced considerably in recent decades, many aspects of their underlying causes and mechanisms remain elusive. Genetic predisposition, environmental influences, and ageing—all contribute to the onset and progression of these diseases [3]. Drug discovery for NDDs is a complex process faced with numerous challenges that slow down the development of effective treatments [1]. One of the most significant challenges is the high failure rate in clinical trials. Since 2003, 98% of treatments for AD, which showed promising results in the preclinical trials, stumbled in the crucial Phase III [4]. This results in significant financial losses and delays in delivering new treatments, which leads to AD remaining incurable [5].

One explanation for this failure may be the reliance on animal models in preclinical drug trials for NDDs, even though such models have shown limited ability to mimic human pathophysiology of NDDs [6]. Creating alternative in vitro models that closely resemble the human body can improve the outcome of clinical trials for new treatments. In vitro testing helps identify the impact of drugs and any associated risks, potentially reducing the time and cost of bringing new treatments to market. For industrial applications, the model’s evolution needs to be continuously and automatically monitored. Conventional methods for in vitro characterisation, including microscopy, immunohistochemistry and enzyme-linked immunosorbent assays (ELISA), provide valuable information on cellular structure, morphology, and specific protein expression. However, microscopy is generally static and does not provide essential information about dynamic changes or cellular responses in real-time. Immunohistochemistry and ELISA provide specificity in detecting biomolecules but require many steps and reagents to perform, lacking the possibility of in situ monitoring or automatization, as well as the opportunity for reuse [7,8,9].

One of the promising technologies for drug discovery is in vitro models with integrated biosensors. A biosensor is an analytical device that uses biological components, such as enzymes or antibodies, among others. It is closely associated with a transducer, converting a biological signal into an electrical signal. The highly specific interactions between these components and the analyte enable the qualitative or quantitative detection of biochemical parameters in complex samples [10]. Biosensors offer real-time, sensitive detection of molecular and cellular changes and provide immediate feedback on disease progression and drug efficacy or toxicity [11,12]. Furthermore, electrochemical biosensors offer the same type of fabrication methods as microfluidics and electrical circuits, making them easily integrable, miniaturisable and automatable at low cost. Moreover, the potential for biosensors’ reusability makes this technology more commercially competitive [13,14].

To enhance our understanding of NDDs and diagnose them on time, innovative biomarkers and tools are required [15,16,17,18]. Neurofilaments, especially neurofilament light (NfL) polypeptide, have gained significant attention as a non-invasive and accessible biomarker found in biofluids. NfL plays a crucial role in maintaining the structural integrity of neurons and is involved in various cellular functions. At the early stage of neuronal degeneration, NfL is released into the cerebrospinal fluid (CSF) and then into the bloodstream [19]. While other biomarkers are associated with specific neurodegenerative pathologies, NfL serves as a general marker of axonal damage, making it applicable across a wider range of NDDs [19,20,21,22,23]. Another distinguishing feature of NfL is its dynamic nature, which allows monitoring disease progression and treatment response [24]. A rapid increase in NfL after axonal damage makes it an early indicator of disease progression, which is crucial for analysing the pathology onset [25]. The NfL utility goes beyond diagnosis, and its use as a biomarker in in vitro and in vivo models can transform the way we monitor NDD models. Despite numerous reported models successfully detecting various biomarkers with electrochemical biosensors, only a few reports use the potential of NfL. The biosensor developed by Özgür et al. uses a porous polymeric film with glycidyl methacrylate for anti-NfL antibody immobilisation [26]. Although this method shows high sensitivity (with the limit of detection (LOD) at 5.21 pg mL^−1^), it requires over 14 h of preparation and has not been tested in a real matrix. Valverde et al. reported a magnetic microbead-based electrochemical bio platform for the NfL determination [27]. The system relies on sandwich-type immunoassay complexes with horseradish peroxidase (HRP) attached to generate an electroactive product, enabling amperometric detection. With the discovered LOD at 3.0 pg mL^−1^, short analysis time (60 min) and easy preparation, this platform is promising but does not mention the use of the cell culture samples.

In this context, we have developed an electrochemical biosensor for NfL detection to analyse acute neuronal injury in an in vitro model of human neurons derived from long-term neuroepithelial-like stem (lt-NES) cells. The biosensor gives a closer observation of drug-induced acute neurotoxicity, potentially useful for drug testing in pharmaceutical research of an NDD’s progression. It was designed in two configurations: a label-free immunosensor based on cyclic voltammetry (CV) and electrochemical impedance spectroscopy (EIS) readout, and a sandwich immunoassay with a secondary antibody with redox labelling, analysed by chronoamperometry (CA). First, we evaluated the biosensor’s ability to detect NfL in controllable standard samples, focusing on the sensitivity and reproducibility of its responses. Once optimised, we assessed its ability to detect NfL in real samples of lt-NES neuronal cultures. Our results suggest that such immunosensors could be used to monitor neurodegeneration in in vitro models of NDDs, providing real-time evaluation with a low-cost readout and a potential for automation.

## 2. Materials and Methods

### 2.1. Reagents

11-Mercaptoundecanoic acid (MUA), (11-mercaptoundecyl)tetra(ethylene glycol) (MUTEG), N-(3-dimethylaminopropyl)-N-ethylcarbodiimide (EDAC), N-hydroxysuccinimide (NHS), diethanolamine, 3,3′,5,5′-Tetramethylbenzidine (TMB) Liquid Substrate System for ELISA (peroxidase substrate), TWEEN^®^ 20, MES hydrate, Bovine serum albumin (BSA), Phosphate-buffered saline (PBS), Potassium nitrate (KNO_3_), Potassium hexacyanoferrate(II) trihydrate (K_4_Fe(CN)_6_·3H_2_O), Potassium hexacyanoferrate(III) (K_3_Fe(CN)_6_), Sodium carbonate (Na_2_CO_3_), Sodium bicarbonate (NaHCO_3_), Polyvinylpyrrolidone (PVP), Poly-L-ornithine hydrobromide, Cyclopamine, Paraformaldehyde, Triton™ X-100, Fluoromount™ Aqueous Mounting Medium were obtained from Sigma-Aldrich (St. Louis, MO, USA). Nunc^®^ MaxiSorp™ 96-well microtiter plate, SuperBlock™ Blocking Buffer, Gibco™ Laminin Mouse Protein, Hoechst 33342, Goat anti-Mouse IgG, IgM, IgA (H+L) Secondary Antibody, Alexa Fluor™ 488 (A-10667) were supplied by Thermo Fisher Scientific Inc. (Waltham, MA, USA). BrainPhys™ Neuronal Medium was provided by STEMCELL Technologies (Vancouver, BC, Canada). Sulfuric acid (H_2_SO_4_) and Hydrochloric acid (HCl) were obtained from Panreac Química SLU (Barcelona, Spain). Screen-printed gold electrodes (SPEs) were supplied by Metrohm DropSens (Oviedo, Spain). Recombinant Human 68kDa Neurofilament/NF-L protein (His tag) (NfL; ab224840), Anti-68kDa Neurofilament/NF-L antibody [EP675Y] (Ab_1_; ab52989), Anti-68kDa Neurofilament/NF-L antibody [DA2] (Ab_2_; ab7255) were provided by Abcam Limited (Cambridge, UK). Anti-mouse IgG, HRP-linked Antibody (Ab_3_–HRP; #7076) was supplied by Cell Signaling Technology, Inc. (Danvers, MA, USA). Staurosporine was purchased from Selleckhem (Houston, TX, USA). N-methyl-D-aspartate (NMDA) was obtained from Tocris Bioscience (Bristol, UK). NF-light^™^ (Neurofilament light) Serum ELISA kit was provided by UmanDiagnostics AB (Umeå, Sweden). Long-term neuroepithelial-like stem (lt-NES) cells were derived from the human induced pluripotent stem (iPS) cells and produced in the Laboratory of Stem Cells and Restorative Neurology (Lund University, Sweden) as described [28]. Human iPS cells were obtained from Prof. Gianvito Martino (San Rafaelle Vita-Salute University, Italy).

### 2.2. Methods

#### 2.2.1. Characterisation Using the ELISA Technique

##### Nfl Antibody-Antigen Characterisation by ELISA

The antibodies used for the biosensor construction were first optimised by ELISA, according to the following protocol. Each step was performed at room temperature (RT, 20–23 °C), unless stated otherwise. The volumes are specified for each well. After each incubation, wells were washed by adding three times 300 μL of 0.05% TWEEN^®^ 20 in PBS (pH 7.4), then discarding it, gently tapping the plate against absorbent paper.

The total of 60 μL of capturing antibody (Ab_1_) in coating buffer (0.05 M Na_2_CO_3_, pH 9.5) was plated on a Nunc^®^ MaxiSorp™ 96-well plate overnight at +4 °C. Remaining protein binding sites were blocked with 100 μL of 1% BSA in PBS for 30 min, followed by a 60 min incubation with 60 μL of NfL in PBS. Next, 60 μL of detection antibody (Ab_2_) in MES buffer (0.05 M, pH 5.0) was added for 30 min, and then 60 μL of anti-IgG antibody conjugated with HRP (Ab_3_–HRP) in 1% BSA/PBS, for 30 min. The HRP was detected by 100 μL of TMB/H_2_O_2_ substrate. The reaction was allowed to proceed in the dark for 15 min before it was stopped by adding 100 μL of 0.18 M H_2_SO_4_. The absorbance was measured at 450 nm (reference wavelength was set at 650 nm) using an ELISA plate reader (Infinite^®^ M200 PRO Multimode Microplate reader, Tecan, Männedorf, Switzerland).

The checkerboard titration was performed by subsequently diluting the reagent directly in the plate. For this, during each titration, each row or column (depending on the direction of titration) was filled with the dilution buffer. Then the reagent in the initial concentration was introduced to every well of the first row/column, mixed thoroughly with the buffer, then transferred to the next row/column, mixed, and so on. The last row and column only had a buffer and were considered blank.

Chosen concentrations of Ab_1_ and Ab_2_ were subsequently re-optimised directly on SPEs by systematically varying each reagent and monitoring the response at fixed NfL concentrations to account for differences in mass transport and surface area.

##### ELISA as a Control Standard Technique

The NF-Light^TM^ Serum ELISA kit was employed as a standard technique for NfL detection for comparison purposes. The steps were performed as described by the manufacturer. The absorbance was measured at 450 nm (reference wavelength was set at 650 nm) using an ELISA plate reader. A fitted four-parameter logistic model was used to generate the calibration curve; the blanks were included as zero.

#### 2.2.2. Biosensor Fabrication

All the electrochemical measurements were carried out using EC-lab software on an electrochemical workstation (version 11.61; BioLogic SP-150, BioLab, Seyssinet-Pariset, France). All following steps with SPEs were performed at RT, unless stated otherwise. The volumes are specified for each electrode. After each incubation, the electrodes were rinsed with PBS-T followed by MES by pipetting several drops of solution on top of the electrode surface using a Pasteur pipette.

The SPEs were cleaned before functionalisation by repeating voltametric cycles with 0.1 M H_2_SO_4_. The potential was cycled 30 times from 0.2 to 1.5 V at a scan rate of 150 mV s^−1^. Following that, electrodes were rinsed with ethanol and blow-dried gently in a stream of nitrogen.

The self-assembled monolayer (SAM) layer comprised MUA and MUTEG. Each electrode was incubated overnight in 20 µL of a mixture containing 1 mM MUA and 0.25 mM MUTEG in absolute ethanol in specially designed chambers to avoid evaporation. Afterwards, electrodes were rinsed with ethanol and gently blow-dried in a stream of nitrogen. The MUA’s carboxylic acid groups were activated during a 15 min exposure to 20 µL of a mixture containing 400 mM EDAC and 100 mM NHS. The generated amide was reacted with the IgG antibody’s amino group by incubating 10 µL of 1.6 µg mL^−1^ Ab_1_ in MES for 60 min. Unreacted amides were blocked with 15 µL of 1 M diethanolamine for 30 min. A 10 µL volume of either blank or an NfL-spiked sample was introduced for 45 min.

To fabricate the labelled sandwich immunosensor configuration, 10 µL of the second antibody (Ab_2_), at 2.5 µg mL^−1^ in 1% BSA/PBS, was introduced for 30 min. This step was followed by a 30 min incubation with 10 µL of Ab_3_–HRP, diluted 50 times in 1% BSA/PBS.

Finally, biosensor regeneration was conducted by placing 20 µL of 1 M hydrochloric acid (HCl) for 10 min on the working electrode surface, followed by the rinsing procedure with PBS-T and MES.

The regeneration efficiency was evaluated through the residual activity, adapted from Amine et al. [29] (Equation (1)):

(1) $$Residual\;activity=\;\frac{I_{after}(C)}{I_{before}(C)}\;\times \;100\%,$$

where *I_before_* and *I_after_* are the CA current values at 10 s before and after the regeneration, respectively. During this experiment, the regeneration was performed after each detection of 1 µg mL^−1^ NfL in the sandwich configuration in order to assess the ability of the biosensor to recover its analytical response in subsequent measurements.

#### 2.2.3. Electrochemical Characterisation of the Immunosensor

The characterisation of each layer during biosensor assembly and the target detection was done using CV and EIS. For this, 20 µL/electrode of a diffusional redox mediator K_3_[Fe(CN)_6_]/K_4_[Fe(CN)_6_] (5 mM in 1 M KNO_3_, pH 6, 1:1 ratio) was placed on the electrode surface. The CV scans were conducted with two replicates at the rate 50 mV s^−1^, where the potential cycled from −0.3 to +0.3 V. The peak values of the second cycle were collected for the data analysis.

The EIS scans were performed over a range of frequencies from 0.5 Hz to 200 kHz, using a modulation voltage of 10 mV with an amplitude of 10 mV versus Ag/AgCl reference. The data were visualised as Nyquist plots and fitted in a Randles circuit with the ZView^®^ software (version 4.0; Scribner, LLC, Southern Pines, NC, USA).

For the HRP detection in the labelled biosensor configuration, CA technique was conducted with 20 µL/electrode of TMB/H_2_O_2_ substrate at 0.2 V for 15 s. The current values at 10 s mark were collected.

#### 2.2.4. Neuronal Cell Culture

##### Cell Culture and Collection of Conditioned Media

Next steps were performed in a sterile laminar flow hood. Human cortical neurons were sourced from iPSC-derived lt-NES cells, as mentioned earlier. First, human skin fibroblasts were taken from a healthy donor, then the fibroblasts underwent a retroviral transduction to yield iPSCs. These iPSCs were then guided through a differentiation process to adopt a neural phenotype, involving the formation of embryoid bodies to produce neural rosettes. The rosettes were meticulously selected, isolated, and cultured in the presence of neuronal-promoting factors to generate lt-NES cells. Subsequently, a differentiation protocol, employing three small molecules, 10 ng mL^−1^ bone morphogenetic protein 4 (BMP-4), 10 ng mL^−1^ wingless-type MMTV integration site family, member 3a (Wnt-3a) protein, and 1 mM cyclopamine, was followed for 7 days to induce the development of cortical neuronal progenitors from lt-NES cells.

The obtained cortical neuronal progenitors were cultured in 24-well pre-coated plates in BrainPhys™ medium supplemented with 2% B27 (without vitamin A; BP). Beforehand, 0.1 mg mL^−1^ poly-ornithine in distilled water was incubated in the plates overnight at RT, followed by thorough washing with distilled water. A total of 5 µg mL^−1^ laminin in PBS was then added to the wells and incubated for at least 2 h at 37 °C/5% pCO_2_. Neuronal progenitors (250,000, 150,000 and 100,000 cells per well) were seeded after laminin removal and kept at 37 °C/5% pCO_2_ for 7 days. The medium was refreshed every two days.

At day 7, 10 µM NMDA or 1 µM staurosporine—both in BP—was incubated with the neuronal culture for 30 min or 24 h, respectively, to cause cell death and induce NfL release through axonal degeneration. Media samples were collected before drug administration (0) and at various time points after (10 min, 60 min, 24 h). After each collection, the medium was replenished, maintaining the drug’s concentration.

##### Imaging and Immunostaining

Neuronal culture on 2D-coverslips were used for imaging and immunostaining to examine the morphological development and confluency of the neuronal cultures. On day 7, representative brightfield (BF) images were captured to examine the density and network formation before fixation, using a microscope (IX71, Olympus Corporation, Tokyo, Japan) equipped with a camera (C10600-10B-H, Hamamatsu Photonics, Hamamatsu City, Japan). Images were acquired using a 20× objective. All the images were further analysed and processed using Fiji software (version 1.54p; open-source software distributed under the GNU General Public, ImageJ [30]).

On the same day, the 2D neuronal cultures were fixed with 4% paraformaldehyde in PBS for 15 min at RT. All following steps were performed at RT, unless stated otherwise. Following fixation, fixed cells were washed three times with 1.5 mg mL^−1^ glycine in PBS (PBS-gly), each time the solution was incubated for 5 min before discarding to remove residual fixative. To allow antibody access to the cytoskeleton, cells were permeabilised with 0.1% Triton™ X-100 in PBS-gly for 10 min. Non-specific binding was blocked using 3% BSA in 0.1% Triton™ X-100/PBS (T-PBS) for 1 h. Samples were incubated overnight at 4 °C with a primary antibody against NfL (Ab_2_, mouse anti-NfL, 1:500) in 3% BSA/T-PBS. Following three PBS washes with a 5 min incubation each, cells were incubated for 2 h with an Alexa Fluor™ 488-conjugated secondary antibody (goat anti-mouse, 1:1000) in 1% BSA/T-PBS. The excess was removed, and the cells were rinsed three times with PBS with a 5 min incubation each. Nuclei were counterstained with Hoechst 33342 (1:2000) in PBS for 10 min. Finally, cells were rinsed three times with PBS with a 5 min incubation each and the cells were maintained in PBS. Coverslips were mounted over a glass slide using Fluoromount™ medium and left to dry overnight at RT. Cells were observed by confocal microscopy (LSM 800, Zeiss, Jena, Germany) using a 20× objective. To maintain consistency for quantification, all fluorescent images were acquired using identical exposure times and gain settings.

#### 2.2.5. Data Processes and Analysis

For each biosensor, raw electrochemical responses (CV peak current of the second cycle, EIS semicircle value, and CA current value at 10 s) were collected and normalised to the measurement obtained immediately before sample incubation (to the baseline, *S_norm_*), or in the case of CA, following the Ab_2_ incubation to account for variations in electrode surface area and initial conductivity.

For CV and EIS, the low-concentration response was approximately linear. Hence, LOD was calculated using the standard 3.3 σ/m, according to the ICH Q2(R1) approach [31], where *σ* was the pooled standard deviation of small-concentration responses and *m* was derived from the linear portion of the calibration curve. For CA, the sensor exhibited a sigmoidal response due to enzymatic amplification. LOD was therefore estimated from the 4-parameter logistic (4PL) fit by identifying the concentration corresponding to the signal equal to the blank mean plus *3σ*. This method accounts for both baseline noise and the non-linear response characteristic of enzyme-mediated detection.

To estimate which responses could be pooled, for each experiment we assessed whether signals at the lowest concentrations were significantly different from the blank using a one-sample *t*-test. If the responses were not statistically different, they were pooled to estimate baseline variability for LOD calculations.

The working range (WR) was determined independently for each electrochemical method (CV, EIS, and CA) based on their specific signal characteristics. For CV measurements, the WR was defined from the lowest concentration above the LOD, exhibiting a coefficient of variation (%CV) ≤ 20% up to the highest concentration before reaching signal saturation or increased variability. For EIS and CA measurements, where higher inter-electrode variability was observed, the WR was defined based on concentrations showing a consistent monotonic response and sufficient sensitivity to changes in analyte concentration, rather than strictly applying a %CV-based criterion. In all cases, signals were evaluated across electrodes, and statistical significance relative to the baseline was assessed using non-parametric Kruskal–Wallis tests with post hoc pairwise comparisons.

Matrix effect of near low-end samples was assessed by measuring low-concentration NfL samples in the cell culture media. First, raw electrochemical signals were normalised to their respective baseline values (*S_norm_*). Subsequently, the net signal change (Δ*S_Matrix_*) was determined by subtracting the blank (0 pg mL^−1^) response. Matrix effects were quantified by comparing responses in both matrices for each method at several concentrations (C) using the following formula (Equation (2)):

(2) $$Matrix\;effect\left(C\right)=\;\frac{{\Delta S}_{BP}(C)}{{\Delta S}_{PBS}(C)}\times 100\%$$

where Δ*S_BP_*(*C*) and Δ*S_PBS_*(*C*) are the averaged signals in BP and PBS, respectively. Unknown concentrations were interpolated using a piecewise linear regression model.

Data analysis, graphing and visualisations were performed using GraphPad Prism (version 10.4; GraphPad Software, San Diego, CA, USA) and Affinity Designer (version 3.0; Serif, Affinity by Canva, Nottingham, UK).

## 3. Results

### 3.1. Immunosensor’s Configurations and Optimisation of the Conditions Using ELISA

**Figure 1:**
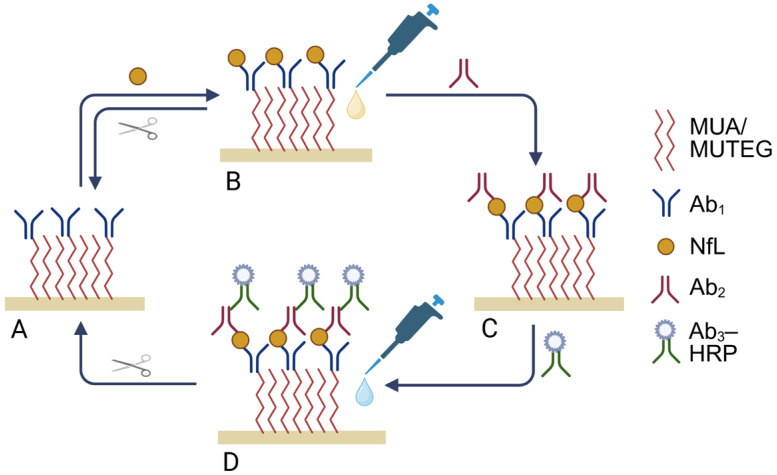
Schematic representation of the biosensor’s configurations and its reusability cycle. (**A**) A label-free system that detects neurofilament light (NfL) in a sample. Capture antibody (Ab_1_) is attached to the gold surface of the electrode via self-assembled monolayer (SAM) (consisting of 11-mercaptoundecanoic acid (MUA) and (11-mercaptoundecyl)tetra(ethylene glycol) (MUTEG)), represented by red lines. (**B**) NfL detection is confirmed by the cyclic voltammetry (CV) and electrochemical impedance spectroscopy (EIS) techniques in ferro-/ferricyanide mediator (represented by a yellow drop). (**C**) Detection antibody (Ab_2_) is added to form another configuration. (**D**) A sandwich system with anti-IgG antibody conjugated with HRP (Ab_3_–HRP). The chronoamperometry (CA) technique in TMB/H_2_O_2_ substrate (represented by a blue drop) is used to detect NfL. Configurations B and D can be reversed to the initial state A by introducing 1 M HCl for 10 min (represented by scissors) that breaks the bond between Ab_1_ and NfL.

The proposed biosensor platform employs two configurations, schematically presented in Figure 1. The first one is a label-free immunosensor that includes the capture antibody (Ab_1_) against the 543rd amino acid in the C-terminal site of human NfL (Figure 1A), which directly detects the antigen in the sample. The characterisation of each layer and the target detection are done with CV and EIS.

The second proposed configuration is a sandwich immunoassay that additionally incorporates the detection antibody (Ab_2_) against 441–455 amino acids in the same site of human NfL and the secondary antibody conjugated with HRP (Ab_3_–HRP), used for detecting antigen–antibody interaction (Figure 1D). The CA technique was chosen for the indirect detection of NfL through the Ab_3_–HRP attachment to the electrode.

A quick identification of the optimal concentrations and conditions required a reliable time- and cost-effective technique, such as ELISA. The chessboard titration technique was selected to optimise the concentration of all three antibodies involved due to its capability for the simultaneous successive dilution of two reagents in each experiment.

The working concentration of NfL was chosen based on the reported working range of commercial ELISA kits: 100 ng mL^−1^. The range of concentrations of the Ab_1_ and the Ab_2_ was chosen according to the general recommendations: 1–12 and 0.5–5 µg mL^−1^, respectively. The recommended dilution for the Ab_3_–HRP was taken from the manufacturer’s note (1000 times).

The first trial on ELISA confirmed a high level of repeatability (within the range specified by the manufacturer, 2.9–5.9%); thus, it was decided to run every condition in one replicate for a quick screening. Finally, optimised conditions were run in duplicates to confirm the results.

**Figure 2:**
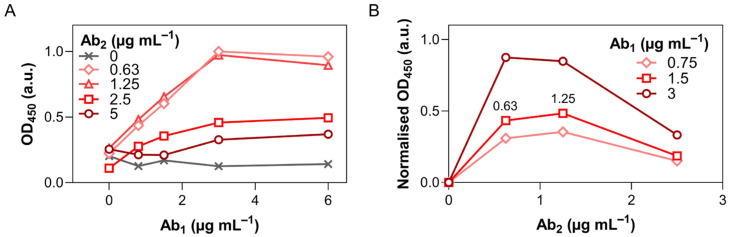
Calibration curves of Ab_1_ and Ab_2_ obtained by enzyme-linked immunosorbent assay (ELISA). (**A**) OD_450_ response for two-fold serially diluted Ab_1_ concentrations (6.00, 3.00, 1.50, 0.75, 0.00 μg mL^−1^) at five Ab_2_ concentrations (5.00, 2.50, 1.25, 0.63, 0.00 μg mL^−1^). (**B**) Normalised OD_450_ response for the range of Ab_2_ concentrations (0.00–2.50 μg mL^−1^) at three Ab_1_ concentrations (0.75, 1.50, 3.00 μg mL^−1^). Values are background-subtracted using the corresponding Ab_2_ = 0.00 signal at each Ab_1_.

To identify optimal concentrations, we evaluated OD_450_ responses across a matrix of Ab_1_ and Ab_2_ concentrations by simultaneously titrating both in a two-fold serial dilution, while fixing NfL and Ab_3_–HRP concentrations (Figure 2A). Signal increased with Ab_1_ concentration, peaking around 3.00 µg mL^−1^, in the 0.63–1.25 µg mL^−1^ range of Ab_2_. At higher Ab_2_ levels, a drop in signal was observed, which may be attributed to a hook effect, steric hindrance, or non-specific interactions interfering with sandwich formation. To better visualise Ab_2_’s influence, a reversed plot of normalised-to-the-background OD_450_ responses vs. Ab_2_ concentration for several Ab_1_ concentrations (0.75, 1.50, 3.00 µg mL^−1^) was generated (Figure 2B). The OD_450_ peaked reliably at 0.63–1.25 µg mL^−1^ Ab_2_. Even though the test produced a higher signal at 3.00 µg mL^−1^ Ab_1_ across the whole range of Ab_2_, we aimed at minimising reagent waste and non-specific signal, hence proceeding with 1.50 µg mL^−1^ Ab_1_ paired with 1.25 µg mL^−1^ Ab_2_, making it a cost-effective and robust solution.

It is worth noting that the OD_450_ in the blank control wells (Figure 2A, grey crosses) was not consistent and relatively high, suggesting inadequate blocking of the Ab_2_ and/or Ab_3_–HRP and resulting in non-specific adsorption on the microwell surface. Originally, Ab_2_ was diluted in MES buffer, and Ab_3_–HRP in 0.1% BSA/PBS. To decrease non-specific adsorption, these and other solutions, including 1% BSA, 0.1% TWEEN^®^ 20, 1% PVP,—all diluted in PBS—and SuperBlock™ Blocking Buffer were evaluated as potential buffers to reduce non-specific binding (Supplementary Figure S1). The final selection was in favour of 1% BSA/PBS. This solution provided sufficient blocking capabilities without simultaneously covering and passivating the electrode surface and interfering with the electrochemical signal.

For similar reasons, the washing buffer, used to rinse electrodes, was changed from 0.1% BSA/PBS to MES buffer solution.

### 3.2. Electrochemical Immunosensor Fabrication and Further Optimisation

Demonstrated in Figure 1 is a schematic overview of the biosensor configurations used for NfL detection: a label-free (Figure 1B) and a sandwich (Figure 1D) system. In the label-free configuration, NfL was directly detected in the sample by Ab_1_. The presence of NfL on the sensor surface interferes with the redox signal of a diffusional mediator (K_3_[Fe(CN)_6_]/K_4_[Fe(CN)_6_]), indicating antigen–antibody binding, which is detected with CV and EIS. In the labelled sandwich system, NfL was detected through its binding to an Ab_2_–Ab_3_–HRP complex. The presence of the sandwich complex, due to interaction with the target, was detected with CA upon addition of TMB/H_2_O_2_, the substrate of HRP conjugated to Ab_3_.

Due to the different surface-area-to-volume ratio and diffusion regime on planar SPEs, we had to re-optimise the concentrations obtained by ELISA. Ab_1_ and Ab_2_‘s concentrations were systematically varied and both signal amplitude and also high-dose hook/steric hindrance effects were analysed. Increasing Ab_1_ from 1.5 µg mL^−1^ (ELISA-derived) up to 6 µg mL^−1^ initially enhanced signal but also promoted early onset of the hook effect at high NfL loads. Thus, we chose the intermediate Ab_1_ concentration of 1.6 µg mL^−1^, for easier diluting process. At the same time, Ab_2_‘s concentration was initially increased to 25 µg mL^−1^, which resulted in a higher but unstable signal. Decreasing it to 2.5 µg mL^−1^, coupled up with the increased Ab_3_–HRP concentration (50 times diluted instead of 1000), improved the electrochemical signal and yielded the best performance on SPEs.

In both configurations, Ab_1_ was linked to the gold surface of the electrode through the SAM (Figure 1). To enhance the antifouling properties of the biosensor, a mixed SAM comprising MUA and a polyethylene glycol (MUTEG in our case) was formed on the electrode surface at a 4:1 molar ratio, respectively, according to the previous works [32,33]. Binary SAMs of MUA with PEG/OEG thiols have been widely used to balance the availability of the carboxyl group for probe immobilisation combined with PEG-based antifouling brushes. The literature suggests that increasing the PEG fraction improves antifouling but reduces probe density and signal amplitude. In this work, we selected a composition of 80 mol% MUA/20 mol% MUTEG to maintain a high density of reactive –COOH groups and a strong analytical signal, while introducing a PEG component to reduce non-specific protein adsorption [34]. This composition provided stable signals and acceptable embedding on our matrix, compared to other variations used in this study and the bare gold electrode. Incorporated MUTEG passivated the surface areas not covered by MUA, reducing background signal and improving sensor-to-sensor reproducibility.

For the characterisation of each immobilised layer during the sensor fabrication process, CV and EIS techniques in 5 mM K_3_[Fe(CN)_6_]/K_4_[Fe(CN)_6_] were employed. A potassium ferro-/ferricyanide diffusional mediator was used to monitor the deposition of molecules on the electrode surface and, therefore, the correct bonding of each layer. Dense coverage of the surface leads to an increase in the electrode surface passivation and to a reduced electron-transfer rate between the electrode and the redox mediator.

**Figure 3:**
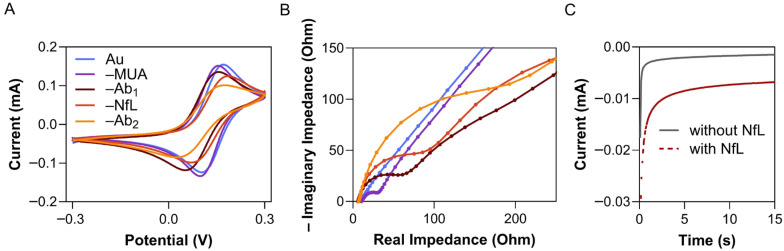
Samples of electrochemical data. (**A**) Cyclic voltammograms and (**B**) impedance spectrograms (Nyquist plots) were obtained during the characterisation of each layer’s immobilisation on the biosensor’s surface with CV and EIS, respectively. (**C**) Chronoamperograms for blank and NfL-spiked samples were generated by CA for a sandwich system.

The voltammograms and spectrograms of each layer, namely, the bare gold (Au) electrode, Au–MUA, Au–MUA–Ab_1_, Au–MUA–Ab_1_–NfL, and Au–MUA–Ab_1_–NfL–Ab_2_, are depicted in Figure 3. Cyclic voltammograms (Figure 3A) are obtained by measuring the current at the working electrode during the potential sweeps: reduction and oxidation processes. They characterise the reversibility of a reaction and the electrode behaviour. Each successive element of the immunosensor assembly—MUA, Ab_1_, NfL, and Ab_2_—further blocks the electrode surface and progressively lowers the peak current in the voltammograms.

The Nyquist plots (Figure 3B) exhibit a semicircle and a linear segment. The semicircular region typically manifests at higher frequencies, signifying the electron transfer process to the electrode surface. Conversely, the linear segment corresponds to processes constrained by diffusion. To ascertain the impedance values of each layer, the Randles circuit model was employed to fit the experimental data. This model comprised a series of components: the electrolyte resistance (R_s_), the charge transfer resistance (R_ct_), corresponding to the diameter of the semicircle, and a parallel combination of the double-layer capacitance (C_dl_) and the impedance of faradaic processes that define the diffusion-limited mass transport (the Warburg impedance, Z_w_) [35]. Table 1 summarises the fitted results for each new immobilised layer.

Each successive immobilisation step leads to an increase in Rct, the circuit element most sensitive to changes in interfacial thickness. The growing surface coverage progressively impedes electron transfer between the electrode and the ferro-/ferricyanide redox mediator. As seen in the Table 1, R_ct_ was gradually increasing from 2.6 to 108.3 Ω. In turn, the C_dl_ fitted from the Randles circuit decreased from 7.58 µF cm^−2^ for the bare electrode to 2.79 µF cm^−2^ after binding to the NfL. This trend is consistent with increased surface coverage and partial blocking of the electrode/electrolyte interface. The Warburg element, associated with the diffusion of the ferro-/ferricyanide redox probe, was in the range of 679–2677 Ω s^−1/2^. The values were increasing with each immobilised after MUA layer, reflecting on slower diffusion in mass transport at the electrode surface upon its functionalisation.

The goodness-of-fit parameter (χ^2^) was on the order of 10^−3^, indicating a high degree of agreement between the experimental and modelled impedance data. Obtained values are consistent with the ones reported for modified electrode systems [36,37].

Additionally, performance evaluation of the sandwich system was conducted using CA in the TMB/H_2_O_2_ substrate solution (Figure 3C). CA investigates the kinetics of redox reactions, diffusion processes, and adsorption. With NfL present in the sample, both Ab_2_ and anti-IgG antibody, conjugated to HRP, bind to the sensor. Upon addition of the TMB/H_2_O_2_ solution, the HRP label catalyses TMB oxidation, and the electrode reduces the oxidised product, causing the reduction current to become increasingly negative. Opposite to the label-free configuration, the NfL is indirectly detected through the Ab_2_–Ab_3_–HRP complex.

All three techniques were employed during the optimisation process, each offering distinct advantages and limitations. While CV and EIS were both used after each immobilisation step, CV was particularly useful for the initial screening: the voltammogram shape offered instant visual cues about the immobilisation quality, unlike EIS, which required further acquisition and analysis. On the other hand, spectrograms showed clear resistance shifts, offering a more promising route for NfL quantification. Concurrently, CA proved valuable for confirming the system’s proper functioning and ensuring accurate validation of the biosensor’s performance.

### 3.3. Strategy for Biosensor Regeneration

Developing a cost-effective biosensor required making it reusable without affecting the bioreceptor molecules on its surface. To selectively break the hydrogen bond, the van der Waals force between the analyte (NfL) and the antibody binding epitope was carefully weakened during a 10 min introduction of 1 M HCl [38,39,40]. Such a strategy kept the Ab_1_ immobilised and active for at least four more tests.

**Figure 4:**
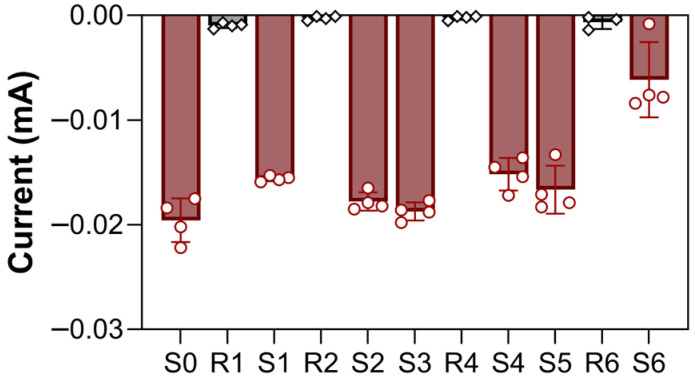
The results of the biosensor’s regeneration cycles. CA was run each detection of an NfL-spiked sample (1 µg mL^−1^) was introduced (*Sn*—response from a sandwich configuration); then, the biosensor was regenerated by adding 1 M HCl for 10 min (*Rn*—biosensor’s response after a regeneration step), followed by a sandwich system formation again. Data are presented as mean ± SD of raw electrochemical responses from independent measurements (*n* = 4).

The regeneration efficiency was evaluated by comparing the CA signal before and after each regeneration step (Equation (1)). Following HCl treatment, the residual current decreased to values close to the baseline level, indicating effective removal of the bound antigen (Figure 4). On average, the chosen strategy demonstrated 98.5 ± 9.8% (*n* = 4) of signal recovery, indicating that the electrochemical response was preserved during repeated regeneration cycles. Stable analytical responses were observed for up to five regeneration cycles. During the sixth cycle, a pronounced decrease in signal was observed (S6), indicating progressive deterioration of the sensing interface.

It is worth mentioning that the regeneration could be performed for both biosensor configurations (Figure 1, regeneration step is represented by the scissors).

### 3.4. Biosensor Performance in the Controlled Matrix

To characterise the developed biosensor, such key analytical parameters as LOD and WR were assessed in the controlled matrix (PBS).

First, standard calibration curves were generated using NfL-spiked samples across a wide concentration range. For CV and EIS, the sensor response is approximately linear at low NfL concentrations, so the LOD was estimated as 3.3*σ*/*m*, where *σ* represents the baseline variability and *m* is the local sensitivity (slope). Across two independent experiments in PBS, CV yielded LODs between 6 and 10 pg mL^−1^. EIS exhibited the lowest analytical sensitivity with LODs ranging from 3 to 6 pg mL^−1^, though it was more susceptible to electrode-to-electrode variability.

In contrast, CA exhibited a sigmoidal response due to the enzymatic amplification, making the low-concentration region non-linear. Therefore, LOD for CA was determined from the 4PL fit. A complete summary of matrix-specific parameters is provided in Table 2.

**Figure 5:**
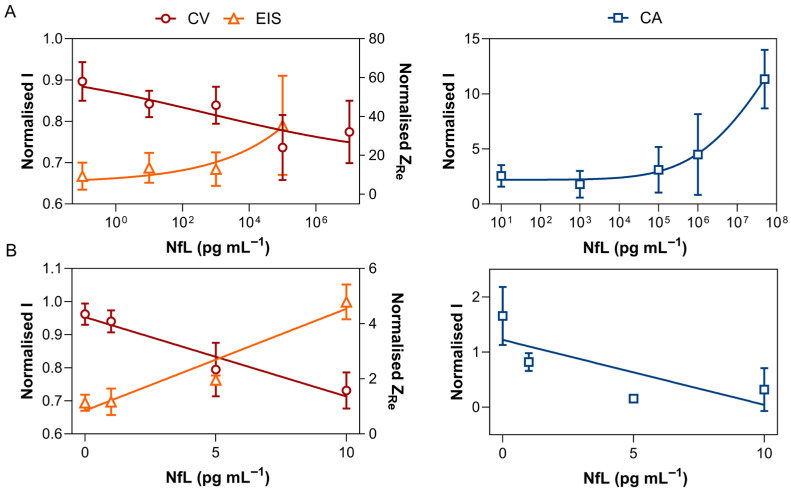
Dose–response curves for the recombinant NfL vs. electrochemical signal. (**A**) Calibration curves in PBS assessed by CV (red circles), EIS (orange triangles), and CA (blue squares). Data are presented as mean ± SD from independent measurements (*n* = 4). (**B**) Linear regression curves in the BrainPhys™ medium supplemented with 2% B27 (BP) obtained from CV (red circles), EIS (orange triangles), and CA (blue squares). Data are presented as mean *S_norm_* ± SD from independent measurements (for CV and CA, *n* = 4; for EIS, *n* = 2). In all, CV data are represented as the peak current (left *y*-axis), EIS, as negative imaginary impedance (right *y*-axis) and CA, as current value at 10 s.

For the label-free configuration (CV and EIS, Figure 5A, left), the WR were defined from 10 to 10^7^ pg mL^−1^ for CV and from 10 to 10^5^ pg mL^−1^ for EIS. CV demonstrated signal saturation beyond 10^7^ pg mL^−1^, characterised by a calibration curve plateau and increased variability. Statistical analysis (Kruskal–Wallis with post hoc testing) identified a significant difference between the signal at 10^5^ pg mL^−1^ and the baseline (*p* = 0.034), suggesting that detectable changes primarily occur at high concentrations under the current conditions. The collected data were normalised (*S_norm_*) prior to analysis.

In the meantime, EIS demonstrated a monotonic increase in signal across the tested range; however, increased inter-electrode variability at higher concentrations limited the reliable working range to 10^5^ pg mL^−1^. Similarly, statistical analysis confirmed the significant difference between the signal at 10^5^ pg mL^−1^ and the baseline (*p* = 0.025), supporting the concentration-dependent response of the system.

In contrast, the CA signal exhibited the characteristic lag phase of an enzymatic response. While low LOD was achieved, the calibration curves showed variability between experiments, with signal magnitude depending strongly on assay conditions. As a result, a consistent working range could not be reliably defined, and CA was evaluated primarily in terms of sensitivity rather than quantitative performance. Statistical analysis confirmed that only the highest concentration was significantly different from the baseline (*p* = 0.023).

To assess the matrix effects of cell culture media on biosensor performance, LODs were first evaluated in BP to simulate the real biological samples’ environment, using the same approach. CV and EIS showed similar LODs of approximately 3 and 4 pg mL^−1^, respectively. In contrast, CA exhibited a pronounced matrix effect; responses were inconsistent and non-monotonic across the tested concentration range. This is likely due to the complexity of media components, which introduce higher background fluctuations and may interfere with the TMB turnover or enzyme–label stability. Thus, the LOD could not be estimated using the same approach within this range of concentrations.

The matrix effect was further evaluated across concentrations of 1–15 pg mL^−1^ by comparing responses in both matrices for each method using Equation (2). Analysis revealed that BP components strongly suppressed early binding signals, particularly for EIS at 1 pg mL^−1^ (2.7% recovery). As NfL concentrations increased to 10 pg mL^−1^, both EIS and CV showed substantial signal recovery, reaching 164.6% and 160.5% of their PBS baseline responses, respectively. This indicates that while the matrix dampens initial sensitivity, the sensor surface remains highly active for quantification at higher NfL concentrations.

In contrast, the enzymatic response of CA was consistently inhibited across the evaluated range, reaching a maximum recovery of only 4.7% at 15 pg mL^−1^. Considering the elevated LOD, the enzymatic amperometric detection is unsuitable for NfL quantification in the cell culture media at the pg mL^−1^ range.

The LOD and WR obtained were noteworthy and close to those observed in the commercially available kits or even surpassed them. For example, one of the most sensitive tests, Multiplex Assay Kit for Neurofilament, Light Polypeptide (NEFL) by FLIA (Flow Luminescence Immunoassay) (CLOUD-CLONE CORP, USA) is claimed to detect NfL below 0.33 pg mL^−1^, while its WR is 0.98–1000 pg mL^−1^. Although the sensitivity is slightly superior, the detection range falls far behind the proposed immunosensor’s WR, making our system more versatile in application. Moreover, FLIA is a high-cost detection method that requires specialised equipment, giving our biosensor an advantage in terms of accessibility and cost-effectiveness.

The developed biosensor focuses on in situ monitoring of biomarkers of neuronal injury directly in the neuronal culture, where NfL levels are typically 1–20 pg mL^−1^. Thus, the sensitivity of our biosensor is well suited for continuous real-time detection of axonal damage in such cultures, allowing applications in neurotoxicity screening and disease modelling. Furthermore, since the presence of NfL in healthy adult is typically 100–1000 pg mL^−1^ in CSF and around 5–10 pg mL^−1^ in serum/plasma, increasing even further in neurodegenerative diseases, the WR of the developed biosensor makes this platform promising for diagnostic use [41].

### 3.5. A Biosensor-Based In Vitro Model for NfL Detection During Neuronal Degeneration

**Figure 6:**
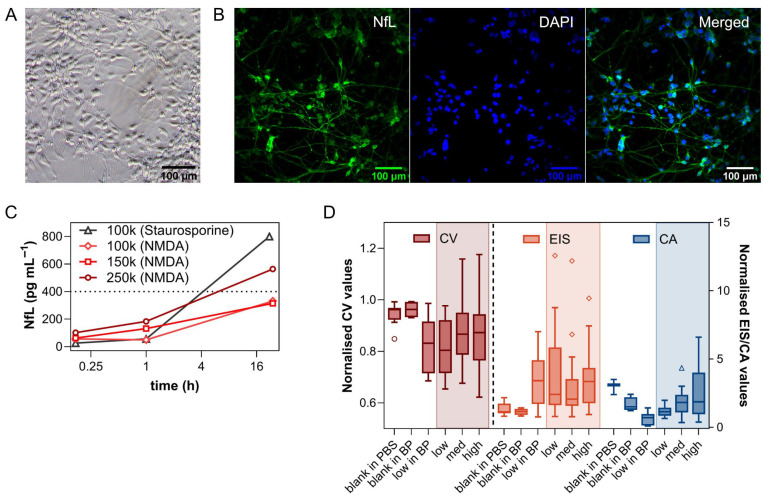
(**A**) A brightfield (BF) image of the neuronal culture on day 7, showing established cellular density and neurite outgrowth. Scale bar = 100 μm. (**B**) Immunofluorescence images of the culture at the same time point. From left to right: NfL (green) highlighting axonal structures; DAPI (blue) indicating nuclear localisation; and a merged overlay. Scale bar = 100 μm. (**C**) NfL concentration in the samples, collected at various time points (10 min, 60 min, 24 h) following drug administration, measured by ELISA. Cells were treated with staurosporine (100k cells per well) or NMDA (100k, 150k, 250k cells per well). Data are represented as the mean. All points beyond 400 pg mL^−1^ of NfL are estimated. (**D**) Tukey’s box plots of the *S_norm_* obtained by CV (red, left *y*-axis), EIS (orange), and CA (blue; both on the right *y*-axis) for blanks in two matrices—PBS and BP—and three groups of cell culture samples (at low, medium, and high concentration) in BP; separated data points were considered outliers.

To evaluate the performance of the developed electrochemical biosensor under real conditions, an in vitro model of acute neuronal damage was used. For this purpose, cortical neuronal progenitors derived from lt-NES stem cells were cultured at several concentrations (250,000, 150,000 and 100,000 cells per well) in BP in a coated 24-well plate for seven days, until neurons were mature. Their maturity was assessed visually by the brightfield (BF) imaging (Figure 6A) and immunofluorescence analysis (Figure 6B). Both revealed the structural integrity of the neuronal network, with NfL-positive axons (green) extending from DAPI-stained nuclei (blue).

To induce axonal damage and neuronal death, we treated the cell with NMDA and staurosporine [42,43,44]. Following the drug administration, the culture samples were collected at 10 min, 60 min and 24 h. At each interval, the entire volume of medium (350 µL) was collected for analysis. Then each well was refilled with fresh BP medium, removing any existing NfL, while maintaining the drug concentration where necessary.

The performance of the biosensor was validated with a standard commercial test, the NF-Light^TM^ Serum ELISA kit test. NfL concentration in the cell culture samples was quantified by this test and then compared to the response of our developed platform. Because the entire medium was replaced at each interval, the NfL concentration showed a non-linear rend at early time points. Specifically, this sampling method accounts for a 16.7% loss of cumulative signal at 60 min, as the first 10 min of production were removed during the initial media exchange. This effect was negligible by the 24 h mark.

There was a clear correlation between NfL concentration, cell density, and time passed since drug administration, according to ELISA results. In general, higher neuronal concentrations and longer waiting time led to NfL levels elevating among all samples (Figure 6C). NMDA produced a rapid NfL rise detectable already at 10 min, consistent with early axonal disruption. While staurosporine generated greater mortality with higher accumulation by 24 h, reflecting delayed apoptotic axonal degeneration, proving to be an effective model of drug-induced acute neurotoxicity. These patterns demonstrate the sensor’s ability to detect mechanistically distinct short-term release profiles

Real biological samples were analysed using the same preparations employed for ELISA quantification to ensure a consistent and directly comparable reference across techniques. As a result, most of the samples evaluated electrochemically were diluted to fall within the ELISA working range (1–25 pg mL^−1^), and the concentration of the rest was extrapolated from ELISA calibration curves (25–50 pg mL^−1^). Much higher NfL levels were not included in this study, focusing on biosensor performance within the ELISA-validated range.

Neuronal cell culture samples produced a highly variable signal. In the lookout for any possible trends, the samples were grouped by low (<12 pg mL^−1^, around the LOD of CV and EIS), medium (12–25 pg mL^−1^), and high (>25 pg mL^−1^; extrapolated) NfL concentration, and presented as Tukey’s box plots; separated data points were considered outliers (Figure 6D). When comparing the results obtained from the neuronal samples (highlighted areas) combined with low-concentration spiked samples in BP, and blanks from both matrices (PBS and BP), a distinct separation between those two groups was noted, especially for CV and EIS. This indicates that even with all the background noise, which could be explained by the complexity of the real samples’ composition, all three detection techniques recognise NfL.

These findings highlight the effectiveness of the biosensor for real-time qualitative detection of neuronal injury in in vitro models, offering a promising, cost-effective tool for studying neurodegenerative models and drug testing.

Looking closer between groups with different NfL concentrations, both CV and EIS showed an inconsistent trend, in contrast to results obtained in PBS and BP medium, where charge transfer resistance increased with concentration in agreement with progressive surface blocking. This may be attributed to extensive electrode fouling caused by cellular debris released during drug-induced neuronal death. The medium contains membrane fragments, intracellular proteins, extracellular vesicles, nucleic acids, metabolites, and lipids, all of which can non-specifically adsorb to the electrode surface, reducing the biosensor’s capacity for a quantitative analysis.

CA measurements proved to be more robust when transitioning from standard samples to real culture media samples showing a clear trend of the mean and maximum current response for these data groups in relation to the NfL concentration, even though these concentrations were below the possible LOD of this technique. These findings highlight that while the biosensor remains responsive for a qualitative analysis in complex media, quantitative analysis is limited due to non-specific adsorption and sample-dependent fouling. However, the electrochemical platform successfully detected NfL with respect to the blanks in real samples and exhibited semi-quantitative behaviour in controlled media (PBS and BP), with pg mL^−1^ level of sensitivity, which is comparable to commercial systems. The performance in the neuron-conditioned medium represents the most challenging measurement scenario, and the current results provide a clear way for future optimisation, in particular, improved antifouling chemistries and regeneration protocols. Among the tested approaches, the combination of CV and EIS on the label-free platform proved most efficient, requiring fewer reagents, less time, and fewer steps.

Broadly, biosensor development for NfL detection remains a largely underexplored area, with few studies published to date. For example, Li et al. reported a carbon-based immunosensor with a modified surface covered with ZrO_2_@La_2_O_3_, which responds to NfL in the range of 0.05–200 ng mL^−1^ with an LOD of 12 pg mL^−1^ in PBS [45]. In contrast to our work, this biosensor characterisation was performed under a controlled buffer matrix. Moreover, ZrO_2_@La_2_O_3_ powder requires a long (>15 h) and complex preparation under high temperatures (up to 900 °C) in N_2_ atmosphere and vacuum, making this test costly. Our proposed immunosensor has simpler preparation steps (requiring mainly an overnight incubation at RT for the SAM deposition and an additional 105 min), quick testing time (nearly 60 min for a label-free configuration and nearly 70 min more for a sandwich system, resulting in less than 3 h of the whole analysis) with similar LOD and wider WR. In another work, Adil et al. developed a biosensor based on reduced graphene oxide SPEs with sandwich-type immunoassay complexes for detecting NfL and achieved the LOD of 390 pg mL^−1^ in buffer solution and 87 pg mL^−1^ in serum medium [46]. However, their study did not investigate sensor reusability. In contrast, our work suggests successful regeneration of the biosensor. In addition, we utilise gold SPEs with enhanced SAM formation, likely contributing to greater sensitivity and broader working range, as gold-SPEs are known for offering superior sensitivity and selectivity, and easy surface chemistry modification, compared to graphite-based electrodes [47].

The novelty of this work lies in the immunosensor’s ability to directly detect NfL in cell culture medium samples collected from human neurons. To the best of our knowledge, no previous studies have reported NfL detection in such samples, highlighting an unmet need and offering new opportunities for the upgrade of in vitro neurotoxicity models.

In summary, the proposed immunosensor offers several advantages over the traditional methods of NfL detection and other reported biosensing models. It requires a simple sample preparation and basic equipment, leading to its versatile application—an in vitro tool for drug testing, diagnosis, and more. Additionally, its reusability provides a significant advantage over single-use ELISA, making it a flexible and efficient detection platform at a lower cost, while still demonstrating a similar LOD.

## 4. Conclusions

A versatile electrochemical immunosensor with two configurations—labelled and label-free—and three electrochemical detection techniques—CV, EIS, and CA—has been developed for the detection of NfL, an effective biomarker of neurotoxicity at the early stage in NDDs models. The developed biosensor demonstrated low LOD in both PBS and BP, up to 9 pg mL^−1^, which is close to the commercial tests’ performance, and wide WR from 10 up to 10^7^ pg mL^−1^, depending on the detection technique. Compared with traditional methods for biomarker detection, its advantages are also in its simplicity and low cost. Most importantly, it can detect NfL in the cell culture medium collected from human-derived neurons.

The electrochemical biosensor technology for NfL detection will enable automated and real-time monitoring of neuronal cultures’ evolution, which can be an excellent tool for drug testing in the pharmaceutical industry, reducing costs and accelerating the discovery of new drugs in NDDs. Future studies will be focused on the incorporation of the developed biosensor into an organ-on-a-chip (OoC) technology for a multifaceted study of a brain-oC platform in situ.

## Figures and Tables

**Table 1:** Summary of EIS fitting in a Randles circuit.

Immobilised Steps	*R_s_* Ohms (Ω)	*R_ct_* Ohms (Ω)	*C_dl_* Farads (F) cm^−2^	*Z_W_* Ω s^−1/2^	*χ* ^2^
Au	6.5	2.6	7.58 × 10^−6^	1158.0	1.85 × 10^−3^
MUA	10.8	18.1	3.32 × 10^−5^	679.3	2.79 × 10^−3^
Ab_1_	6.5	35.0	1.29 × 10^−6^	870.1	2.04 × 10^−3^
NfL	7.7	43.5	2.79 × 10^−6^	1298.0	5.21 × 10^−3^
Ab_2_	7.4	108.3	2.10 × 10^−6^	2677.0	2.48 × 10^−3^

**Table 2:** Analytical characteristics of the NfL biosensor.

Technique	LOD (PBS) pg mL^−1^	LOD (BP) pg mL^−1^	WR (PBS) pg mL^−1^
CV	~8	~3	10–10^7^
EIS	~4.5	~4	10–10^5^
CA	~9	-	-

## Data Availability

The original data presented in the study are openly available in CORA. Repositori de Dades de Recerca at doi:10.34810/data3146.

## Supplementary Materials

The following supporting information can be downloaded at: https://www.mdpi.com/article/10.3390/bios16040212/s1, Figure S1: Comparison of several buffer solutions used for blocking the antibodies and passivating the biosensor surface.

## Abbreviations

The following abbreviations are used in this manuscript:

**Abbreviation**	**Full Term**
Ab_1_	Capture antibody
Ab_2_	Detection antibody
Ab_3_–HRP	Anti-IgG antibody conjugated with HRP
BP	BrainPhys™ medium supplemented with 2% B27
CA	Chronoamperometry
CV	Cyclic voltammetry
EIS	Electrochemical impedance spectroscopy
ELISA	Enzyme-linked immunosorbent assay
LOD	Limit of detection
Lt-NES	Long-term neuroepithelial-like stem
MUA	11-mercaptoundecanoic acid
MUTEG	(11-mercaptoundecyl)tetra(ethylene glycol)
NDDs	Neurodegenerative diseases
NfL	Neurofilament light
SAM	Self-assembled monolayer
WR	Working range
