# Supplementary material for: Electrochemical Detection of Neuronal Injury in Cell Culture Samples: A Cost-Effective Biosensor for Neurofilament Light Sensing
Source: Biosensors (Basel). 2026 Apr 9;16(4):212. doi: 10.3390/bios16040212 (PMC13115547; doi:10.3390/bios16040212)
Supplement: Supplementary file 1 [file biosensors-16-00212-s001.zip › biosensors-4207198-supplementary.pdf]

biosensors

MDPI

Supplementary Materials

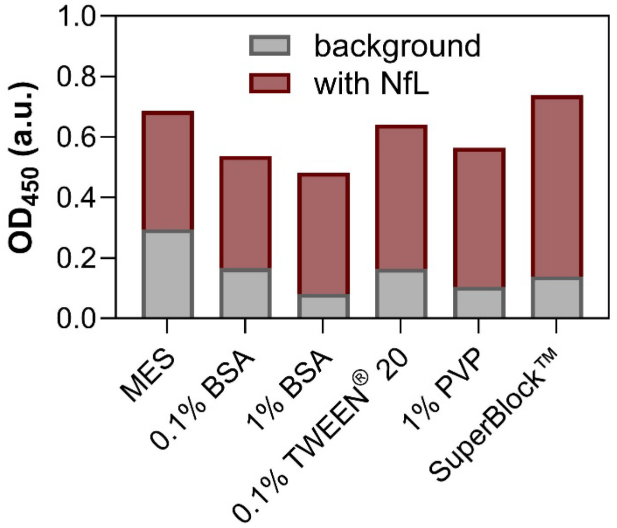

Figure S1. Comparison of several buffer solutions used for blocking the antibodies and passivating the biosensor surface. Data, obtained from ELISA, are presented as combined background signal (blank sample) and signal from an NfL-spiked sample (100 ng mL^{-1}).
